# Precision annotation of digital samples in NCBI’s gene expression omnibus

**DOI:** 10.1038/sdata.2017.125

**Published:** 2017-09-19

**Authors:** Dexter Hadley, James Pan, Osama El-Sayed, Jihad Aljabban, Imad Aljabban, Tej D. Azad, Mohamad O. Hadied, Shuaib Raza, Benjamin Abhishek Rayikanti, Bin Chen, Hyojung Paik, Dvir Aran, Jordan Spatz, Daniel Himmelstein, Maryam Panahiazar, Sanchita Bhattacharya, Marina Sirota, Mark A. Musen, Atul J. Butte

**Affiliations:** 1 Institute for Computational Health Sciences, University of California, San Francisco, California 94158, USA; 2 Department of Neurosurgery, Stanford University School of Medicine, Stanford, California 94305, USA; 3 University of Illinois College of Medicine, Chicago, Illinois 60612, USA; 4 Harvard Medical School Department of Immunology, Harvard University, Boston, Massachusetts 02115, USA; 5 Wayne State University School of Medicine, Detroit, Michigan 48201, USA; 6 Yale School of Medicine, Yale University, New Haven, Connecticut 06519, USA; 7 University of Vermont Medical Center, University of Vermont, Burlington, Vermont 05401, USA; 8 Program in Biological & Medical Informatics, University of California, San Francisco, CA 94158, USA; 9 Stanford Center for Biomedical Informatics Research, Stanford University School of Medicine, Stanford, California 94305, USA

**Keywords:** Data mining, Data acquisition

## Abstract

The Gene Expression Omnibus (GEO) contains more than two million digital samples from functional genomics experiments amassed over almost two decades. However, individual sample meta-data remains poorly described by unstructured free text attributes preventing its largescale reanalysis. We introduce the Search Tag Analyze Resource for GEO as a web application (http://STARGEO.org) to curate better annotations of sample phenotypes uniformly across different studies, and to use these sample annotations to define robust genomic signatures of disease pathology by meta-analysis. In this paper, we target a small group of biomedical graduate students to show rapid crowd-curation of precise sample annotations across all phenotypes, and we demonstrate the biological validity of these crowd-curated annotations for breast cancer. STARGEO.org makes GEO data findable, accessible, interoperable and reusable (i.e., FAIR) to ultimately facilitate knowledge discovery. Our work demonstrates the utility of crowd-curation and interpretation of open ‘big data’ under FAIR principles as a first step towards realizing an ideal paradigm of precision medicine.

## Introduction

The paradigm of precision medicine^1–6^ is based largely on first understanding the genomic features of disease and then designing biomarkers and drugs that identify and rescue these genomic defects respectively. Thus far, precision medicine has gained the most traction in cancer^7^ where for both non-small cell lung cancer and breast cancer, for instance, the standard-of-care now includes sequencing of genes such as EGFR or quantitating panels of RNA such as those included in Oncotype DX, respectively, to drive therapeutic decisions for new subtypes of patients^7^. Moreover, clinical trials are ongoing to develop a precision medicine approach to other diseases such as those that affect the cardiovascular^8–11^ and neuropsychiatric^12,13^ systems among others. In fact, the National Research Council recently affirmed that to realize the practice of precision medicine requires building a molecular taxonomy or nosology from functional gene targets defined across many different diseases^14^. However, the dearth of machine readable public genomics data appropriately curated over a great number of diseases has largely precluded such efforts.

Meanwhile, the National Center for Biotechnology Information (NCBI) Gene Expression Omnibus (GEO) is perhaps the largest of a number of open functional genomics data repositories^15–17^, and GEO data is rich and complete over a great many diseases and phenotypes. There are currently gene expression measurements openly available on over 2 million samples drawn from experiments amassed since the year 2000^18–20^. Funding agencies, such as the National Institutes of Health (NIH), mandates the public sharing this data from functional genomics experiments, and GEO doubles in size every two years on average. To date, over 21,000 PubMed publications have been derived from over 1,000,000 digital samples (see http://STARGEO.org/stats) measured by microarrays, the single largest type of genomic data within GEO^19^. While this data can openly be used to define a precision medicine ideal, GEO itself and almost all other attempts at crowd-curation of sample level annotations have largely failed to embrace the guiding principles to make data generated more findable, accessible, interoperable and reusable (i.e., FAIR principles)^21^ to ultimately enhance the ability of machines and individuals to leverage GEO data for downstream scientific inquiry.

For instance, while GEO itself elicits a basic level of sample curation for contrasts of phenotypes in GEO DataSets, this curation is largely used to visualize gene expression in a given study (or Series), and most importantly DataSet annotations disregard FAIR principles and are not standardized across studies. Similarly, while the Gene Expression Atlas by ArrayExpress^17^ employs a combination of small scale biomedical manual curation by experts with sophisticated text-mining tools to annotate samples with a structured bioontology across studies^22^, their approach is neither open nor embraces FAIR principles and thus cannot scale GEO’s millions of individual samples. As of October 2015, ArrayExpress had annotated 2,330 datasets studying samples in 6,345 differential comparisons across 25 different organisms (http://www.ebi.ac.uk/gxa/release-notes.html). Moreover, the few other crowd curation attempts^23,24^, including some with an interactive meta-analysis portal (http://metasignature.stanford.edu/), have either failed in their scale to annotate GEO and / or failed to embrace FAIR principles that encourage sustained and ever more useful crowd curation.

With this immediate and increasing need to better mine large open data stores to foster new knowledge discovery, the NIH had a Big Data to Knowledge (BD2K) initiative to maximize the use of biomedical big data for individual investigators and the overall research community. Towards this end, we introduce the Search Tag Analyze Resource for GEO (STARGEO.org) as a NIH / BD2K-funded online platform to share open crowd-curation of digital samples. Currently, no large-scale central repository of annotations exists that the biomedical community can leverage to characterize the molecular genomic pathology of disease. STARGEO.org fills that gap by providing a convenient web-based annotation interface to facilitate precise curation of digital samples, as well as an analysis portal to easily generate robust genomics signatures from meta-analysis of the genomics data and crowd curated annotations.

Towards that end, we recruited a small group of up to 10 biomedical students to develop STARGEO.org into a structured functional genomics database of digital samples curated for relevant biological features. Specifically, we targeted graduate biomedical students as a scientific crowd enriched with disease knowledge whose members are most incentivized to learn about disease genomics in preparation for their careers. Indeed, studies have shown that levels of intrinsic motivation far outweigh extrinsic motivation in inducing crowd participation and to maintain precision of task performance^25^. Therefore, we hypothesize that leveraging a small crowd of biomedical graduate students for the curation of biological features with STARGEO.org will result in a precisely annotated dataset of samples that may be used for large-scale translational discovery.

In this work, we demonstrate a rapid crowd-curation of sample annotations across all phenotypes, we report a high precision of annotation among curators to characterize common annotation mistakes, and we demonstrate high and significant biological validity of crowd-curated annotations on open data for characterizing the genomic pathology of breast cancer.

## Results

### STARGEO.org genomic discovery process

**Figure 1:**
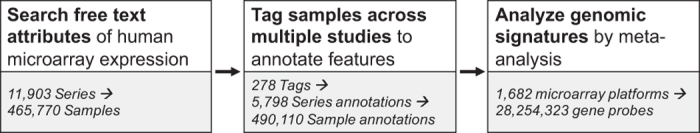
STARGEO.org genomics discovery process. The flow chart shows the three main steps to generate genomic signatures with STARGEO.org. A curator first searches across free text attributes of human microarray expression within GEO. The curator then tags samples across multiple studies to annotate features such as disease status or experimental condition. Finally, users can generate biological genomic signatures of functional gene expression for a given phenotype or experimental condition by meta-analysis.

The general STARGEO.org workflow for using GEO data to define genomic signatures is shown (Fig. 1). Curators first search for free text attributes to apply Tags across multiple studies before they can analyze genomic signatures of disease. For each functional genomics experiment deposited in GEO, a Series provides a focal point and description of the whole study by linking together a group of related Samples. STARGEO.org continually downloads raw data from GEO for all the unstructured free text Sample and Series attributes (defined in the original data deposition by the study authors) for genome-wide human expression profiling by micro-array experiments. We deposit the free text attributes into a database that is indexed to facilitate full text searches of both Samples and Series attributes. This search functionality is built into STARGEO.org, allowing curators to efficiently find specific samples of interest described by specific keywords and modifiers thereby immediately facilitating Findability and Accessibility of raw GEO sample attributes under FAIR principles.

Furthermore, we keep the STARGEO.org data in sync with GEO data, which is continually being updated. Once appropriate studies to curate are found, STARGEO.org curators make annotations on Tags that represents knowledge about digital samples. Specifically, we define Tags and annotations as key:value bindings where Tags are the keys that hold annotation values. We allow users to map Tags to formal ontologies sourced from the National Center for Bioontology’s BioPortal^26^ to immediately make their crowd-curation data Interoperable and machine readable. Also, we provide a snapshot interface for users to quickly assemble and ultimately freeze snapshots of annotations and digitally publish their snapshots to Zenodo (https://zenodo.org) to promote Reusability.

In addition, we automatically map all probe sets or Platforms deposited in GEO to the National Center for Biotechnology Information’s Entrez gene IDs^27^ to allow users to perform robust meta-analyses across Series to define differentially expressed genes. These results we describe here are based on raw GEO data downloaded for 465,770 digital Samples from 11,903 Series (experiments) across 1,682 different Platforms (chipsets) as of December 19, 2013, and we report on 490,110 total sample annotations made on 5,798 series across 278 independent Tags made on that data through December 31, 2015.

### The STARGEO.org curation process

**Figure 2:**
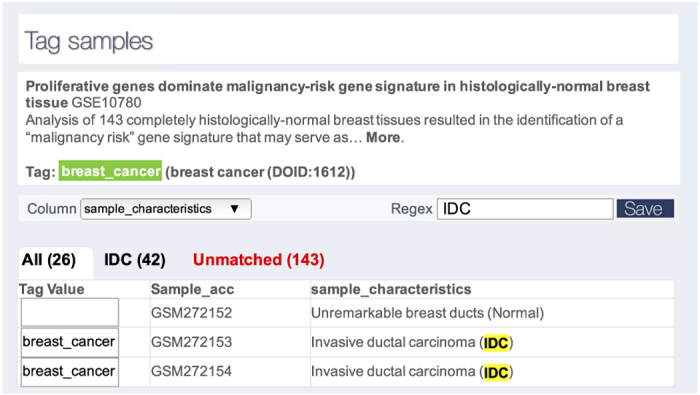
STARGEO.org curation process. The figure shows a STARGEO.org screenshot to annotate the experimental study (GSE10780) entitled ‘Proliferative genes dominate malignancy-risk gene signature in histologically-normal breast tissue’. The Tag has been mapped *a-priori* to the disease ontology and represents a generalized class of breast cancer (DOID:1612). To annotate samples that match to the breast_cancer Tag, the curator selected the sample_characteristics column and designed the ‘IDC’ RegEx to GEO sample descriptors. STARGEO.org automatically highlights matching samples in real-time based on the curator’s RegEx and annotates those samples with the selected Tag. This process is repeated across many different studies and different Tags to explicitly capture all relevant information that is subsequently used to perform meta-analyses.

We implemented the STARGEO.org annotation process (Fig. 2) to allow for manual curation through a simple Tagging interface based on interactive regular expressions (RegExs). Tags define a standardized nomenclature across experiments to represent biological phenotypes such as age, gender, survival, or case or control status of a disease. Specifically, we define Tags as curator-assigned key:value bindings for digital samples where the names of tags are reusable keys that are bound to sample annotation values (for example Age:50, Gender:Female, Cancer:True, etc.). When data is deposited in GEO, the submitter uses specific words or phrases in the raw data attributes to describe contrasts in sample phenotypes. RegExs have long been a standardized syntax in computer science to efficiently match and extract text^28^, and they allow curators to select subsets of Samples in a given Series for mass annotation.

With STARGEO.org, curators design RegExs at the Series level to match and thus discriminate linked Samples in order to assign appropriate Tags. Therefore, a Series with thousands of Samples is Tagged with the same effort as a Series with only ten samples once an appropriate RegEx is used to discriminate Sample level annotations. The web application features real-time highlighting of annotations being applied to samples to make clear the result of any RegEx being applied to Tag samples (Fig. 2). Most of our curators have been able to design RegExs to match Tags that hold Boolean annotations (such as case/control) status. Although, more RegEx savvy users can use parentheses to directly ‘capture’ matching categorical (such as cancer subtype) or quantitative annotations (such as age). In our analysis of the precision of making RegExs below, we find capturing RegEx annotations to be more error prone to simply matching (Boolean) RegEx annotations, and suggest that users should explicitly enumerate Boolean matches for any given set of categorical annotations, and that users only capture quantitative phenotypes.

### Crowd-curation of STARGEO.org annotations

To instantiate the database with high quality annotations, we recruited ten biomedical graduate students from across the country to curate samples for disease and other biological phenotypes, and we designed a reimbursement scheme to reward their precision in making annotations. We used word of mouth and social media to reach out to potential curators. Our sole criterion was that curators had at least some graduate level training in the biomedical sciences. We used Twitter to strategically recruit curators that would be interested in learning about disease and defining genomic disease signatures. Specifically, we sent direct messages to Twitter users with any keywords like ‘biomedicine’, ‘translational medicine’ or ‘research’ in their profile descriptions as well as key words like ‘student’ and ‘MD’ and/or ‘PhD’ to capture their educational exposure. In all, we recruited three different biomedical graduate students from the local Bay area (Stanford and UCSF), and we recruited an additional six biomedical graduate students across the United States with our Twitter outreach.

**Figure 3:**
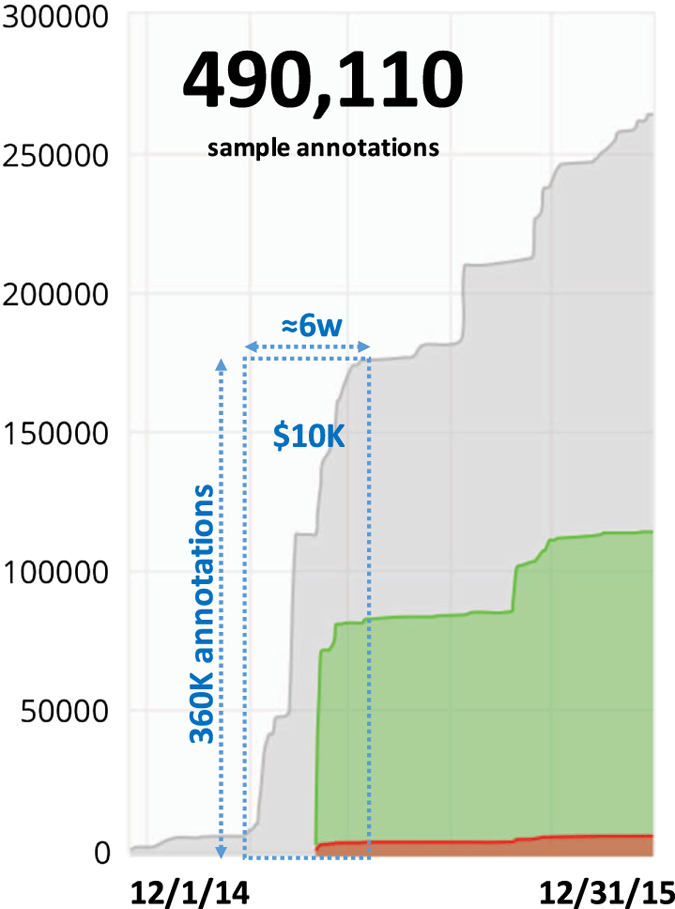
Cumulative distribution of sample annotations. The figure shows the cumulative distribution of 490,110 total annotations collected over a year with >90% concordant (green) and<10% discordant (red) annotations that were performed twice, relative to the sample annotations that were only performed once (grey). Blue dashed box labels the effect of $10,000 reimbursement to yield over 360,000 biological annotations in only 6 weeks.

With this small crowd of curators from 12/1/2014 through 12/31/2015, we made 490,110 total sample annotations using 278 Tags across 149,380 distinct Samples drawn from 11,903 distinct Series. This represents 32% of the 465,770 digital Samples we downloaded from GEO that we have annotated with at least one Tag or 14% of the 1,639 series we downloaded from GEO (Fig. 3). We found our Series level approach to annotating Samples scaled very quickly; in about six weeks we were able to amass over 360,000 individual sample annotations among ten biomedical graduate students. To achieve this rate of coverage, we reimbursed curators to exhaust a total budget of $10,000 during the initial six-week period. We found that this initial reimbursement drove the initial rate of coverage and validation, and once we exhausted our budget, the rate of validation plateaued. Nonetheless, without any reimbursement, some students continued to annotate new samples to define differentially expressed genes and learn about the molecular pathology of disease for their own purposes. Interestingly, the figure also shows a spike in annotations over the summer months independent of any reimbursement as the students had the time and interest to contribute.

Strategically, curators were allowed the freedom to define new Tags in order to represent any phenotype of interest. We employed a text based system to define new Tags to facilitate complete flexibility to describe any biological or experimental feature. In the initial six-week period of reimbursement, virtually all Tags represented disease states except for demographics such as Age, Gender, etc. We manually controlled the vocabulary of Tags by collapsing obvious duplicates (such as ‘Breast Cancer’ and ‘BRCA’) where appropriate. For disease related phenotypes, such as case or control status, we mapped curator-supplied tags to the Disease Ontology^29,30^ *post-hoc* to further standardize the semantic consistency of Tags across studies and to facilitate cohort selection of contrasts for meta-analyses.

### Precision of STARGEO.org annotations

To test the precision in making the 490,110 sample annotations we acquired, we implemented a validation interface for blinded cross-annotation among the curators—i.e., different curators made independent annotations to check the annotation concordance as a measure of precision of already Tagged Samples. We reimbursed pairs of curators 5 cents for every concordant sample annotation to drive precision, and curators were only reimbursed for 100% concordant Sample annotations per Series. To minimize the potential for abuse of our reimbursement scheme and to ensure the highest reliability of our measured cross-annotation precision, we sought to facilitate true independence of the cross-annotations among different curators. Specifically, we hid all GEO identifier fields to completely blind the cross-annotation interface such that curators cannot easily duplicate concordant Sample annotations for a given Series. Similarly, we strategically hide RegExs submitted from users to again discourage automated cross-annotation without manual review.

**Figure 4:**
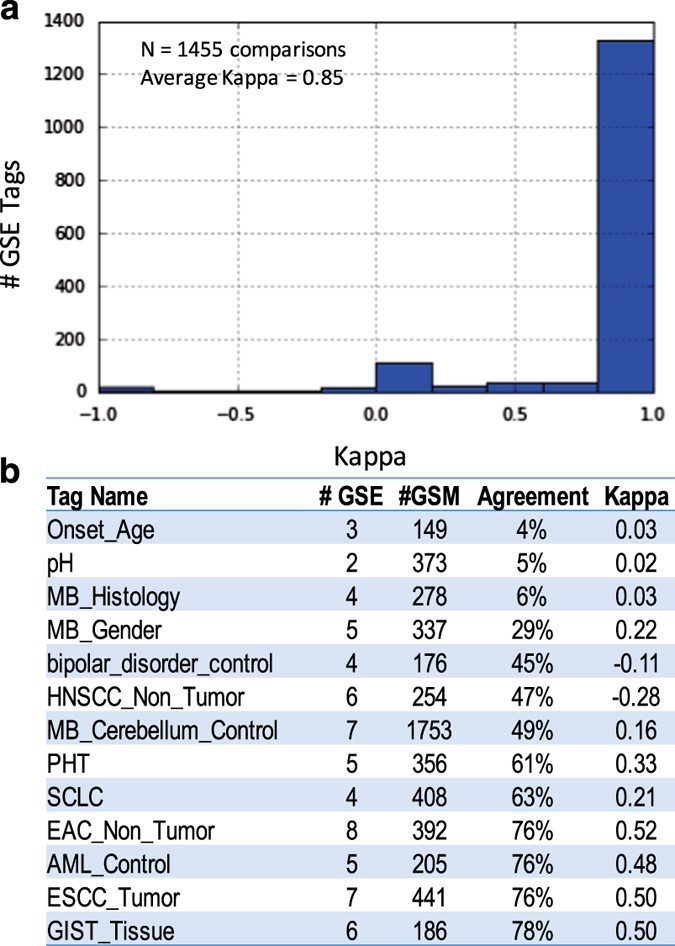
Discordance among cross-annotations. (**a**) The figure shows the distribution of Kappa correlation coefficients of agreement for 1,827 independent pairs of Series containing Samples cross-annotated for the same Tag. The mean Kappa estimate was 0.86, and 81% (1,487/1,827) of the comparisons had perfect Kappa coefficients of 1.0. (**b**) The table shows the most discordant Tags in order of increasing agreement between two independent annotators. The number of contributing Series (#GSE), Samples (#GSM) as well a measure of the agreement and kappa is reported.

With this validation and reimbursement scheme, we made 154,770 distinct annotations across 141 unique Tags (Supplementary File 1) that were blindly validated by an independent curator with an overall concordance rate of 91.1%. These annotations were made on 92,335 distinct Samples drawn from 1,193 distinct Series. Of the 141 distinct Tags that were used, 70% (98/141) were mapped *post-hoc* to bioontology.org covering 84% (130139/154,770) of distinct cross-annotations made (Table 1). As multiple Tags can annotate a given Series, we made 2,084 original annotations at the Series level that were blindly cross-annotated by an independent curator (Supplementary File 2). Cohen’s Kappa coefficient of agreement is a more statistically robust measure of precision than concordance^31–33^, and we estimated Cohen’s Kappa coefficient for 1,827 pairs of Series containing Samples blindly cross-annotated for the same Tag (Fig. 4a). While Samples from the remaining 257 pairs of comparisons at the Series level were highly concordant, Kappa remained undefined because annotations were uniform for each Study without any variability. We found the mean Kappa estimate was 0.86, and 81% (1,487/1,827) of the comparisons had perfect Kappa coefficients of 1.0.

Besides these pairs of annotations sharing perfect agreement, the next most common pattern of agreement centered on Kappa=0, which represents random agreement in 156 comparisons (−0.25<=Kappa<=0.25). We found this random pattern of agreement between pairs of expert annotators involved mistakes in defining RegExs such as with capturing Age, the most frequent phenotype annotated initially and subsequently validated. However, other examples of random agreement involved poorly designed Tags that asked for ambiguous annotations. For instance, one example was the MB_Histology Tag for the GSE21140 Series, which is supposed to represent a histological annotation for medulloblastoma. The original RegEx captured categorical annotations of medulloblastoma histology (RegEx=‘(Classic|Desmoplastic|Large cell anaplastic|MBEN)’). However, the validation RegEx matched on whether the patient had primary medulloblastoma (RegEx=‘Primary medulloblastoma’). When grouped by Tags across multiple Series and Samples, the most discordant tags (Fig. 4b) all derived from either curator mistakes in defining a RegEx to capture a quantitative value (Onset_age and pH) or poorly designed or ambiguous Tags (MB_Histology, MB_Gender).

Additionally, there was a distinct subset of 10 pairs of sets of Sample annotations with perfect disagreement where Kappa=−1. Almost inevitably, these were mistakes made in matching the RegEx for case or control status. For instance, the largest Series with a Kappa=−1 on cross annotation of 144 Samples was for the RCC_control Tag for the GSE53757 Series, which represents control samples for renal cell carcinoma. The original annotation matched samples with normal kidney (RegEx=‘normal kidney’) while the validation annotation matched renal cell carcinoma patients (RegEx=‘clear cell renal cell carcinoma’).

### Validation of STARGEO.org annotations

**Figure 5:**
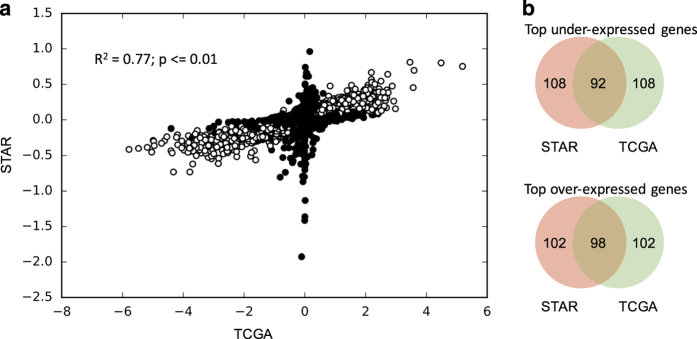
STARGEO.org validation in breast cancer from TCGA. (**a**) The figure shows a significant Spearman rank correlation (*P*<=0.01) of tumor-normal gene effects estimated from STARGEO.org versus TCGA. Circles on the scatter plot shows 19,725 gene effects estimated using 1,119 cases of breast cancer tumors relative to 113 normal breast tissue samples as controls from TCGA (x-axis) and 1,234 cases of breast cancer tumors relative to 535 normal breast tissue samples as controls over 27 different platforms in STARGEO.org. The 3,168 genes that are significant at a false discovery rate of 0.1 after correcting for multiple tests by Benjamini–Hochberg procedure in both TCGA and STARGEO.org are outlined as open white circles, while the remaining genes are drawn as black shaded circles. (**b**) The figure shows the overlap in top 200 (1%) most upregulated and downregulated genes in breast cancer of 19,725 genes estimated among STARGEO.org and TCGA datasets. Red circles represent STARGEO.org genes, green circles represent TCGA genes, and their intersection is beige colored.

To validate the biological accuracy of STARGEO.org annotations, we used The Cancer Genome Atlas (TCGA)^34^ as a gold standard for a well annotated set of samples of functional genomics data, and we compared the rank correlation of the summary statistics for the tumor-normal differential expression of STARGEO.org versus TCGA samples. In particular, breast cancer is the best represented disease among TCGA samples, and we performed differential gene analysis on RNA-Seq data from 1,119 cases of breast cancer tumors relative to 113 normal breast tissue samples as controls. We generated a comparable STARGEO.org measure of differential gene expression for breast cancer with meta-analysis (http://STARGEO.org/analysis/249/) using our crowd-curated annotations. In all, we used 1,234 tumors (cases) versus 535 normal (control) samples of breast tissue over 27 different GEO studies from STARGEO.org. Overall, we found a significant (*P*<=0.01) Spearman rank correlation of 0.77 (Fig. 5a) across all 19,725 gene effects estimated for STARGEO.org and TCGA data, and we found 3,168 genes that are significant at a false discovery rate of 0.1 in both TCGA and STARGEO.org after correcting for multiple tests. Moreover, among the top 200 genes (1%), we found an overlap of 92 most down-regulated versus 98 most up-regulated (Fig. 5b) shared by both STARGEO.org and TCGA analyses. This result is highly significant as an overlap of only two genes is expected by chance.

## Discussion

Robust gene signatures discovered through public disease-related datasets have had tremendous translational impact for biomarker and drug discovery^35^ across transplant rejection^36^, lung cancer^37^, pancreatic cancer^38^, chronic renal disease^39^, preeclampsia^40,41^, and sepsis^42^ among others. However, defining robust gene signatures from public data involves a laborious process requiring substantial technical expertise to download, curate, and analyze digital samples across different datasets. While physicians and scientists are the disease experts most incentivized to annotate and subsequently interpret GEO data, the significant bioinformatics burden to do so precludes their efforts. STARGEO.org immediately solves this problem for individual researchers by providing robust meta-analyzed genomics signatures to users based on their curated annotations of digital samples through the convenient web application. Moreover, STARGEO.org provides a natural mechanism to check those curations for precision and consistency by embracing FAIR principles for crowd curation. This stands in stark contrast of other attempts to annotate GEO, including GEO itself, that disregard FAIR principles thereby handicapping the sustainability of such efforts and development of any robust digital curation community.

In this work, we introduce STARGEO.org as a novel web-based application to gain better descriptions of GEO sample phenotypes uniformly across different studies and to define robust differentially expressed gene signatures of disease by meta-analysis of gene expression. Most importantly, STARGEO.org specifically makes every free text attribute we source from GEO as well as all curation and analysis data we generate immediately FAIR. Moreover, by targeting and reimbursing a specialized crowd of biomedical graduate students, we are able to leverage STARGEO.org to curate biological features with high precision. We found that without any bioinformatics training or experience, the students we recruited were able to dynamically conduct sophisticated meta-analyses to define robust signatures of disease and ultimately discover the molecular pathology of different diseases. As a proof of principle, we demonstrate the biological accuracy of these crowd-curated annotations by significantly recapitulating differentially expressed genes that define breast cancer relative to a TCGA gold standard for a well annotated functional genomics dataset.

We acknowledge that we cannot estimate the performance of Tags to accurately capture crowd-curation of sample annotations for lack of an appropriate gold standard of annotations from the original data depositor. In fact, the gold standard of open data curation is manual review by human curators as we perform here twice with high precision. The high inter-rater reliability we observed among curators suggests that Tags can reproducibly capture the features of biological samples that the original data depositor intended to share. In the absence of an appropriate gold standard, however, it is reasonable to asses curation performance by consensus theory or majority opinion because aggregation of independent responses across curators is more accurate than any individual curator’s response^43–45^, and this relationship is robust and independent of any explicit bias among curators^46^. Therefore, while for lack of a gold standard it remains unclear how sensitive or specific the crowd-curation annotations are, we assume accurate annotations with high inter-rater reliability metrics we demonstrate here despite of any individual curator’s unknown bias.

Finally, STARGEO.org is designed to be for crowd curation of open data what GitHub has been for open source code development: i.e., a community of curators that can openly build large sets of annotations together. Specifically, STARGEO.org is designed to support existing best practices to make research data more findable, accessible, interoperable and reusable (i.e., FAIR principles) to ultimately facilitate knowledge discovery. We embrace FAIR principals for both the crowd-curated sample annotations we generate and the raw sample attribute free-text data that we download from GEO. By making raw GEO data Findable and Accessible, we immediately provide a valuable tool beyond the standard search interface that GEO provides. By building in ontology-mapping functionality from bioontology.org to map our Tags, we immediately make or crowd-curation data Interoperable. We provide a snapshot interface for users to quickly assemble and ultimately freeze snapshots of annotations and digitally publish their snapshots to Zenodo.org (https://zenodo.org) to promote Reusability. Therefore, by adopting FAIR principles, we may transform STARGEO.org into a translational community resource that can be used to capture open digital curation to characterize the functional genomics of disease on a large scale towards discovery of novel drugs and biomarkers in this age of precision medicine.

## Methods

Using the Amazon Web Services cloud infrastructure, we downloaded over 1.7 TB of public data for all processed expression data and associated attributes for series, samples, and platforms catalogued in GEO (ftp://ftp.ncbi.nih.gov/pub/geo/DATA/), and we developed a scalable database schema to represent their attributes as Tags defined as curator-assigned key:value bindings for digital samples where key sample tags are bound to sample annotation values (for example Age:50, Gender:Female, Cancer:True, etc.) on an open-source PostgreSQL (https://www.postgresql.org) relational database management system backend. With this schema, we implemented a web application in Python (https://www.python.org) programming language using the Django (https://www.djangoproject.com) web development framework that allowed us to crowd-curate a semantic network of Tags and appropriate sample annotations representing biological diseases and other phenotypes. We also implemented the functionality for users to quickly assemble and ultimately freeze snapshots of annotations on STARGEO.org and digitally publish their annotation datasets to Zenodo.org (https://zenodo.org) for formal citation.

For the data behind the web application described here, we filtered GEO for ‘expression profiling by microarray’ in humans to find 465,770 digital Samples from 11,903 Series (experiments) across 1,682 different Platforms (chipsets) as of December 19, 2013, and we report on curations made on this raw GEO data through 12/31/2015. We full text indexed all 14,874,580 sample and 283,883 series attributes to facilitate rapid searches at the sample attribute level, a task currently impossible on GEO. We leveraged regular expressions (RegExs) in Python to design an annotation interface for curators to use to quickly annotate sample with Tags to represent biological interpretation. We integrated a blinded validation scheme that allowed for cross-annotation of Tags on which we derived measurements of precision. We used simple concordance estimates as well as Cohen’s Kappa statistic^33^ to measure precision on annotations on blind cross-annotation by independent curators. Additionally, we mapped all microarray probe identifiers to Entrez gene^27^ identifiers using the mygene.info^47^ community annotation service. Finally, we designed a simple analytical interface where more advanced curators could design, compute and visualize standard genomic meta-analysis^48^ of random and fixed effects across tagged and annotated digital samples.

We used STARGEO.org to define a genomic signature for breast cancer on crowd-curated and compared it with a genomic signature for breast cancer using TCGA data. We used STARGEO.org mappings, based on the mygene.info^47^ gene annotation service, to map all probe identifiers to Entrez gene identifiers. For STARGEO.org, we used samples with crowd-curated annotations that were made across 1,234 cases versus 535 control samples from 27 different GEO experiments. For every gene measured in each study, we estimated the mean difference of contrasts for expression as well as the standard deviation of that mean difference. We used a standard meta-analysis with 1) fixed and 2) random effects model to combine these estimates across studies to generate meta P-values and meta effects across studies. Specifically, we used inverse variance weighting for pooling of the data across studies, and calculated weights for estimates of random effects with continuous outcome data via the DerSimonian-Laird estimate^49^. We use Python to implement these analyzes in STARGEO.org. All raw GEO data, curations, and analyses that we generate are available at the http://STARGEO.org web application portal with documentation for programmatic download via a representational state transfer (ReST) application programmer interface (API) through http://STARGEO.org/docs.

For TCGA, we downloaded RNA-Seq data already preprocessed to transcript counts across genes and deposited in GEO with clinical annotations from thousands of samples from TCGA and matched controls (GSE62944). We selected 1,119 breast cancer cases versus 113 controls and performed two standard types of analyses to define differentially expressed genes: (1) A statistical *T*-test based on fragments per kilobase per million sequenced reads (FPKM) estimates^50^, and (2) differential gene expression analysis based on the negative binomial distribution (DESeq2) method^51^. We used Spearman rank correlation across all four comparisons of differentially expressed genes between STARGEO.org (random versus fixed effects) meta-analyses and TCGA (FPKM versus DESeq2) analyses. Although all the comparisons were both highly and significantly correlated by Spearman rank correlation, we found that the highest correlation of the STARGEO.org breast cancer genomic signature under random effects for STARGEO.org and the FPKM for TCGA, and these results are reported as our results. To correct significance for multiple tests, we applied the Benjamini–Hochberg procedure^52^ and selected genes with false discovery rate (FDR)<0.1 (10%). For both STARGEO.org and TCGA analyses, we scaled the fold change of each gene’s effect by the significance (−log10(*P*-value) × fold change), and used this score to rank genes by their differential expression and estimate the overlap among the top 200 (1%) of genes^53^ shared between the two datasets. All calculations are provided as Supplementary Data (Supplementary File 3).

## Additional information

**How to cite this article**: Hadley, D. *et al.* Precision annotation of digital samples in NCBI’s gene expression omnibus. *Sci. Data* 4:170125 doi: 10.1038/sdata.2017.125 (2017).

**Publisher**’**s note**: Springer Nature remains neutral with regard to jurisdictional claims in published maps and institutional affiliations.

## Supplementary Material

Supplementary File 1

Supplementary File 2

Supplementary File 3

## Figures and Tables

**Table 1:** Cross-annotations mapped to ontologies.

**Acronym**	**Description**	**# Tags**	**# annotations**
DOID	Human Disease Ontology	85	49,411
EFO	Experimental Factor Ontology	2	70,875
EO	Ethnicity Ontology	1	42
MEDLINEPLUS	MedlinePlus Health Topics	4	932
MESH	Medical Subject Headings	1	638
SNOMEDCT	SNOMED CT	5	8,241
Unmapped		43	24,631
Grand Total		141	154,770
The table shows the distribution of 154,770 distinct cross-annotations across 141 distinct Tags mapped *post-hoc* to bioontology.org. The table lists the official bioontology.org acronym (accessed through https://bioportal.bioontology.org/ontologies/[Acronym]), the given ontology description, the number of distinct Tags mapped to the given ontology, and the corresponding number of distinct sample cross-annotations.			
